# Compressed Sensing Photoacoustic Imaging Based on Fast Alternating Direction Algorithm

**DOI:** 10.1155/2012/206214

**Published:** 2012-12-30

**Authors:** Xueyan Liu, Dong Peng, Wei Guo, Xibo Ma, Xin Yang, Jie Tian

**Affiliations:** ^1^Sino-Dutch Biomedical and Information Engineering School, Northeastern University, Shenyang, Liaoning 110004, China; ^2^Life Sciences Research Center, School of Life Sciences and Technology, Xidian University, Xi'an, Shaanxi 710071, China; ^3^The College of Electronic Information and Control Engineering, Beijing University of Technology, Beijing, Hebei 100124, China; ^4^Intelligent Medical Research Center, Institute of Automation and Chinese Academy of Sciences, Beijing, Hebei 100190, China

## Abstract

Photoacoustic imaging (PAI) has been employed to reconstruct endogenous optical contrast present in tissues. At the cost of longer calculations, a compressive sensing reconstruction scheme can achieve artifact-free imaging with fewer measurements. In this paper, an effective acceleration framework using the alternating direction method (ADM) was proposed for recovering images from limited-view and noisy observations. Results of the simulation demonstrated that the proposed algorithm could perform favorably in comparison to two recently introduced algorithms in computational efficiency and data fidelity. In particular, it ran considerably faster than these two methods. PAI with ADM can improve convergence speed with fewer ultrasonic transducers, enabling a high-performance and cost-effective PAI system for biomedical applications.

## 1. Introduction 

As an emerging biomedical imaging technique, photoacoustic imaging (PAI) has experienced considerable growth in the past decade [1]. It has been explored for molecular imaging of biomarkers [2], functional imaging of physiological parameters [3, 4] and the imaging of tumor angiogenesis [5–7] in both preclinical and clinical studies. Photoacoustic tomography (PAT) provides speckle-free imaging with high contrast and high resolution which is one form of PAI. When biological tissues are irradiated by short laser pulses, some optical energy is absorbed and converted into heat. The resultant thermoplastic expansion leads to the emission of ultrasonic waves which are acquired by a single-element focused ultrasonic transducer with mechanical scanning or an ultrasonic transducer array from a full view [8, 9]. Then the information of the tissue's optical absorption properties can be recovered using a reconstruction algorithm.

Many algorithms have been developed to exactly or approximately reconstruct the image with a full view of data [10]. A limiting factor for the traditional filtered back-projection (FBP) algorithm is the great number of measurements made with transducers, implying long acquisition times. In addition, it is almost impossible to cover the entire surface of the tissues in many practices. To the best of our knowledge, there is no exact formula reported for limited-view PAT yet. To resolve such limiting factors, based on the compressive sensing (CS) theory [11] can be used to achieve artifact-free imaging from limited-view acquisition.

An image can be reconstructed from far fewer measurements than what the Shannon sampling theory requires if the image is sparse or can be compressed [12]. By using data from a small number of angles and an L1magic convex optimization algorithm, Provost et al. introduced the CS theory into the field of PAT [13, 14]. The issue of artifacts and loss of resolution in limited-view imaging can be addressed by using random optical illuminations for fast data acquisition via the SPGL1 algorithm [15, 16]. Sun et al. have developed an arc-direction compressed-sensing PAT algorithm with numerical phantoms [17]. Both phantom and *in vivo* results showed that the CS method can effectively reduce undersampling artifacts via the nonlinear conjugate gradient descent algorithm [18, 19]. All of these studies have shown that CS-based reconstruction techniques can reduce the number of ultrasonic transducers of the PAT system significantly and obtain high-resolution results with limited-view photoacoustic data. However, one of the critical issues that used to hinder the application of CS in PAT is the computational cost of the underlying image reconstruction process.

In this paper, we proposed a fast CS reconstruction algorithm to overcome this difficulty, leading to acceptable computational times. We studied the use of the alternating direction method (ADM) for L1-norm minimization compressive sensing problems arising from sparse PAT reconstruction [20]. The proposed algorithm was used to improve the speed of the reconstruction from highly incomplete data [21, 22]. The numerical simulation results showed that the ADM algorithm was efficient and robust. In particular, the ADM can generally reduce relative errors faster than all of the other tested algorithms.

## 2. Method

### 2.1. Photoacoustic Imaging

According to the photoacoustic signal generation theory, the acoustic pressure *p*(*r*, *t*) at location *r* and time *t* in an acoustically homogeneous medium obeys the following wave equation [23]:

(1) $$\begin{array}{c} \left(\nabla^{2}-\frac{1}{c^{2}}\frac{\partial^{2}}{\partial t^{2}}\right)p\left(r,t\right)=-\frac{\beta }{C_{p}}\frac{\partial }{\partial t}H\left(r,t\right), \end{array}$$

where *c* is the sound speed, *p* is pressure, *β* is the isobaric volume expansion coefficient, *C*_*p*_ is the specific heat, and *H*(*r*, *t*) is the heating function that can be written as the product of the initial absorbed optical energy density *A*(*r*) and a temporal function of illumination *I*(*t*). If the pulse pumping can be regarded as a Dirac delta function *I*(*t*) = *δ*(*t*), the following problem can be solved using the Green's functions to obtain the pressure:

(2) $$\begin{array}{c} p\left(r,t\right)=\frac{\beta }{4\pi C_{p}}∭A\left(r^{\prime }\right)\delta \left(t-\frac{\left|r-r^{\prime }\right|}{c}\right)\frac{d^{3}r^{\prime }}{\left|r-r^{\prime }\right|}. \end{array}$$

Taking the Fourier transform on variable *t* of (2) and denoting *k* = *ω*/*c*, the forward problem in the temporal-frequency domain is expressed as

(3) $$\begin{array}{c} \bar{p}\left(r,k\right)=\frac{-ick\beta }{4\pi C_{p}}∭A\left(r^{\prime }\right)\frac{\exp\left(ik\left|r-r^{\prime }\right|\right)}{\left|r-r^{\prime }\right|}d^{3}r^{\prime }. \end{array}$$

To numerically model the previously mentioned problem we used a vector *x* to represent *A*(*r*) and a vector *y* to represent the detected acoustic pressure $\bar{p}(r,k)$. Then (3) can be expressed as *y* = Φ*x*, and the forward projection matrix Φ in the temporal-frequency domain can be written as

(4) $$\begin{array}{c} \Phi_{\left(m,n)(i,j\right)}=-ick_{n}\frac{e^{ik_{n}|r_{m}-r_{ij}|}}{\left|r_{m}-r_{ij}\right|}, \end{array}$$

where *m* indicates the position of the transducer, *n* represents the sampling point in the frequency domain, and *r*_*ij*_ indicates the Cartesian coordinates of the image pixels. 

### 2.2. CS Application in PAI

Mathematically, the projection matrix Φ is ill conditioned if the measurement is insufficient. This will lead to uncertainties during the reconstruction. Fortunately, the CS theory tells us that a sparse signal can be exactly reconstructed from incomplete datasets if satisfying some requirements. It has been proven that photoacoustic image is sparse or compressed enough in a certain domain [13, 15]. By finding an appropriate sparse transform Ψ : Ψ*x* = *θ*, the photoacoustic image can be reconstructed by solving a convex optimization problem in the following form [12]:

(5) $$\begin{array}{c} \min{||\theta ||}_{1}\;\text{s}.\text{t}.\;\;y=\Phi \Psi^{-1}\theta . \end{array}$$

When *y* contains noise, or *x* is not exactly sparse but only compressible, as in most practical applications, the constraint in *y* = ΦΨ^−1^ *θ* must be relaxed, resulting in the constrained basis pursuit denoising problem

(6) $$\begin{array}{c} \underset{\theta }{\min}{||\theta ||}_{1}\;\text{s}.\text{t}.\;\;{||y-\Phi \Psi^{-1}\theta ||}_{2}^{2}<\varepsilon , \end{array}$$

where *ε* is the noise level. From the optimization theory, problem (6) is equivalent to the following problem with a suitable parameter:

(7) $$\begin{array}{c} \underset{\theta }{\min}{||\theta ||}_{1}+\frac{1}{2\mu }{||y-\Phi \Psi^{-1}\theta ||}_{2}^{2}. \end{array}$$

As *ε* and *μ* approach zero, both problem (6) and (7) converge to problem (5).

### 2.3. Reconstruction Method

Based on the classical ADM technique, the first-order primal-dual algorithm that updates both primal and dual variables at each iteration was used. With an auxiliary variable *r* problem (7) is clearly equivalent to

(8) $$\begin{array}{c} \underset{\theta }{\min}{||\theta ||}_{1}+\frac{1}{2\mu }{||r||}_{2}^{2}\;\text{s}.\text{t}.\;\;y=\Phi \Psi^{-1}\theta +r. \end{array}$$

Equation (8) has an augmented Lagrange subproblem of the form

(9) min⁡θ,r⁡||θ||1+12μ||r||22−λT(ΦΨ−1θ+r−y)   +β2||ΦΨ−1θ+r−y||2,

where *λ* is a Lagrange multiplier and *β* > 0 is a penalty parameter. Given (*θ* ^*k*^, *λ* ^*k*^), the minimization of (9) with respect to *r* is given by

(10) $$\begin{array}{c} r^{k+1}=\frac{\mu \beta }{1+\mu \beta }\left(\frac{\lambda^{k}}{\beta }-\left(\Phi \Psi^{-1}\theta -y\right)\right). \end{array}$$

For *r* = *r* ^*k*+1^ and *λ* = *λ* ^*k*^ are fixed, the minimization of (9) with respect to *θ* is equivalent to

(11) $$\begin{array}{c} \underset{\theta }{\min}{||\theta ||}_{1}+\frac{\beta }{2}{||\Phi \Psi^{-1}\theta +r^{k+1}-y-\frac{\lambda^{k}}{\beta }||}_{2}^{2}. \end{array}$$

The solution of (11) can be given explicitly by one-dimensional shrinkage

(12) θk+1=Shink(θk−τgk,τβ)=max⁡⁡{|θk−τgk|−τβ,0}θk−τgk|θk−τgk|.

Finally, with a constant *γ* > 0 we updated the multiplier *λ* by

(13) $$\begin{array}{c} \lambda^{k+1}=\lambda^{k}-\gamma \beta \left(\Phi \Psi^{-1}\theta^{k+1}+r^{k+1}-y\right). \end{array}$$

In short, ADM applied to (7) produces the iteration:

(14) rk+1=μβ1+μβ(λkβ−(ΦΨ−1θ−y)),θk+1=Shink(θk−τgk,τβ),λk+1=λk−γβ(ΦΨ−1θk+1+rk+1−y),

where both the primal and the dual variables are updated at each and every iteration.

## 3. Results and Discussion

To demonstrate the efficiency and superiority of the ADM algorithm in PAI reconstruction with limited angle observations, computer simulations were conducted in 2D where the imaged sources are approximately located within the transducer focal plane. All of the experiments were performed using MATLAB (MathWorks, Natick, MA, USA).

Although the existing CS algorithms provided accuracy results, the computational cost of the optimization process was significantly higher, hindering practical application. By using the ADM algorithm, in the following section, the problem with the computational cost of the image reconstruction could be overcome, leading to acceptable reconstruction computational times.

### 3.1. Reconstruction from Simulated Limited-View Data

**Figure 1:**
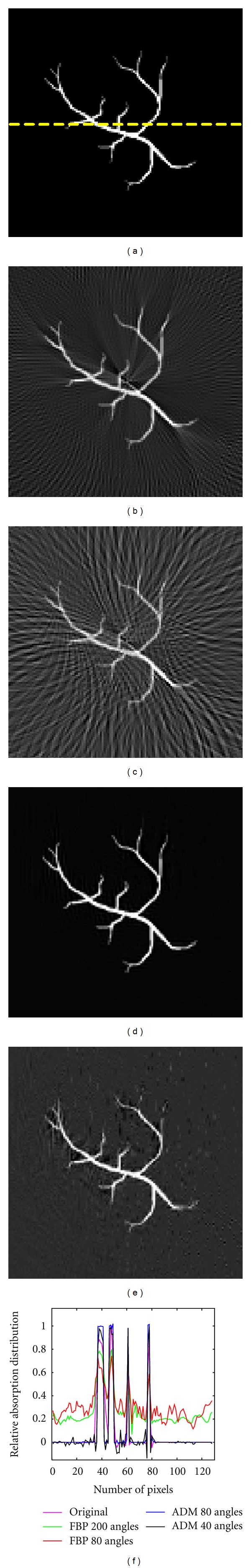
Image reconstructions using the FBP method and ADM algorithm. (a) Original phantom. (b) and (c) image reconstructions using the FBP method with 200 and 80 transducers evenly covering the circle. (d) and (e) image reconstructions using the ADM method with 80 and 40 transducers uniformly covering the 90-degree view. (f) Center lines extracted from (a) to (e).

In this section, based on both the Symmlet wavelet with an order of 4 and L1-regularization, the numerical experiments have been conducted on a sparse 30 mm × 30 mm phantom (shown in Figure 1(a)) with a 128 × 128 resolution. In each experiment, the single-element focused ultrasonic transducer was used to record the photoacoustic signals. To model the transducer response, the domains of *k*_*n*_ and *n* were restricted to certain values *k*_*n*_/2*πc* ∈ [0.2,2.5] and *n* ∈ [1,128]. At every detection angle, 64 randomly chosen *k*_*n*_/2*πc*'s inside the [0.2,2.5] MHz window were used to completely define Φ_(*m*,*n*)(*i*,*j*)_. By rescaling the intensity values of the phantom to [0,1], we generated measurements *y* using Φ_(*m*,*n*)(*i*,*j*)_ in the frequency domain.

The results reconstructed by the FBP and ADM algorithms based on the multiangles observation with a different detection position are shown in Figures 1(b)-1(c) and Figures 1(d)-1(e), respectively. Figures 1(b) and 1(c) show image reconstruction using the FBP algorithm with measurements along a horizontal circle, stopping at the 200 and 80 positions. Figures 1(d) and 1(e) show the reconstruction results using the ADM algorithm with 80 and 40 transducers uniformly covering the 90-degree view. It is shown that the results of the CS method are clearly superior to those of the FBP method. This can be seen by extracting and comparing lines from the reconstructed images in Figure 1(f).

### 3.2. Comparison of CS Reconstruction Algorithms

We compared ADM with L1magic and SPGL1 for the solution model (6). For all of the experiments, the same number of transducers and Fourier samples per angle uniformly covering the 90-degree view was used. In order to compare these three algorithms in a way that is as independent as possible, the same iteration stopping criteria *δ* = ||*x* ^*k*+1^ − *x* ^*k*^||/||*x* ^*k*^|| < 0.005 were used. The quality of the reconstructed image including the number of iterations, the CPU times, and the signal-to-noise (SNR) achieved by each of the algorithms is presented in Table 1; all of which are the average values over 10 runs for each experiment.

We can conclude from Table 1 that there are large differences between the algorithm execution times: ADM can be roughly 6 times faster than SPGL1, which itself is about 20 times faster than L1magic. The proposed algorithm is not only faster, but also maintains the best SNR ratio.

**Figure 2:**
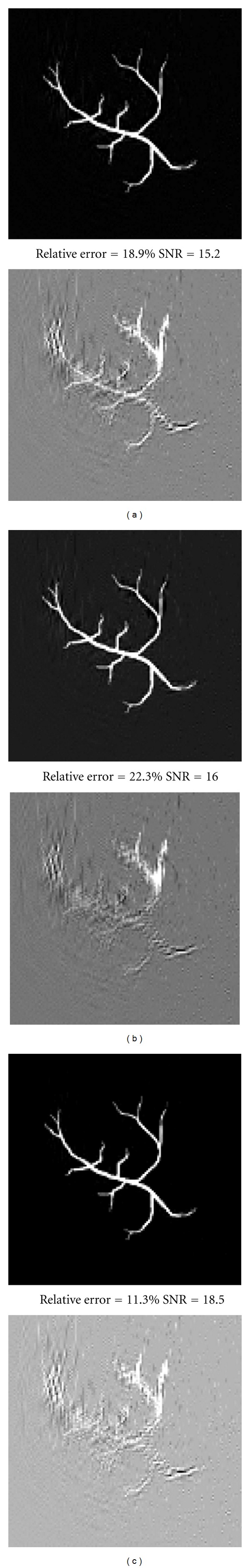
Image reconstructions in (a) with L1magic, (b) with SPGL1, and (c) with ADM, using 56 detection angles and 64 Fourier samples per angle with a uniform distribution at 90-degree curve noisy observation with SNR = 40 dB.

In order to demonstrate the data fidelity of the ADM algorithm, Figure 2 shows the reconstruction results obtained with these three minimization schemes with measurements from 56 detection angles polluted by average SNRs of 40. In terms of reconstruction quality, all of the algorithms produced similar results overall. The denoising effect of the CS can be observed on the residual images. For a computed solution *x*′, its relative error to *x* is defined as

(15) $$\begin{array}{c} \text{RelErr}\left(x\right)=\frac{||x^{\prime }-x||}{||x||}\times 100\%. \end{array}$$

### 3.3. Discussion

**Figure 3:**
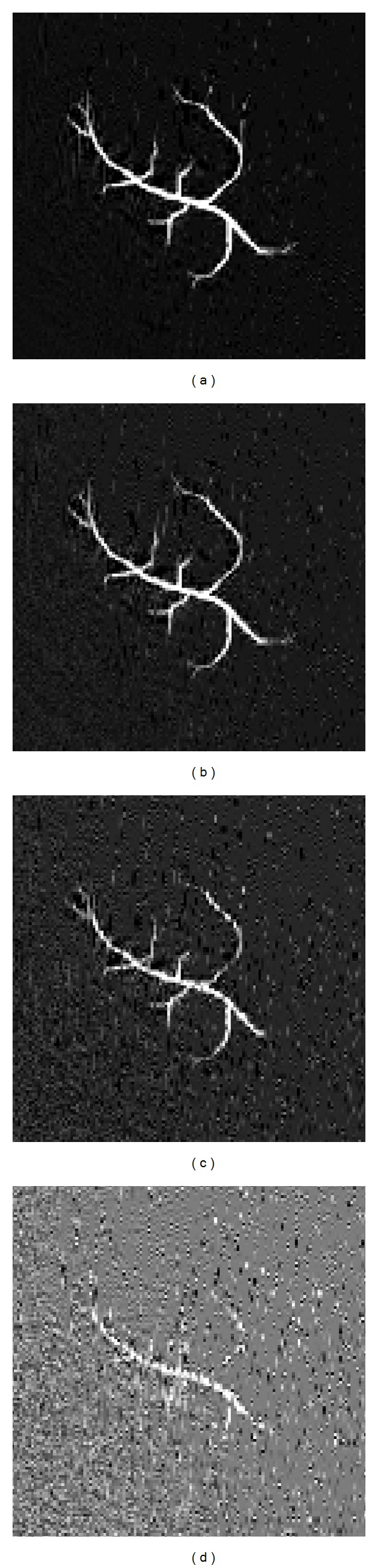
Image reconstructions using the ADM algorithm with 40 detection angles that were uniformly distributed at a 90-degree curve (a) noiseless observation; noisy observation with (b) SNR = 30 dB; (c) SNR = 20 dB; (d) SNR = 10 dB.

It has been demonstrated that 90 degrees is sufficient for high-quality reconstruction. To study the effects of white Gaussian noise polluted measurements on the reconstruction performance for the ADM algorithm, acquisitions were simulated of different SNRs. The reconstructions are shown in Figure 3. As predicted in theory [12, 21], the proposed algorithm based on the CS scheme is robust to inaccurate measurements. By contrasting the influence of different noise levels on the reconstruction results, we can see that the increased measurement noise only increases the reconstruction noise. No major artifacts can be observed.

**Figure 4:**
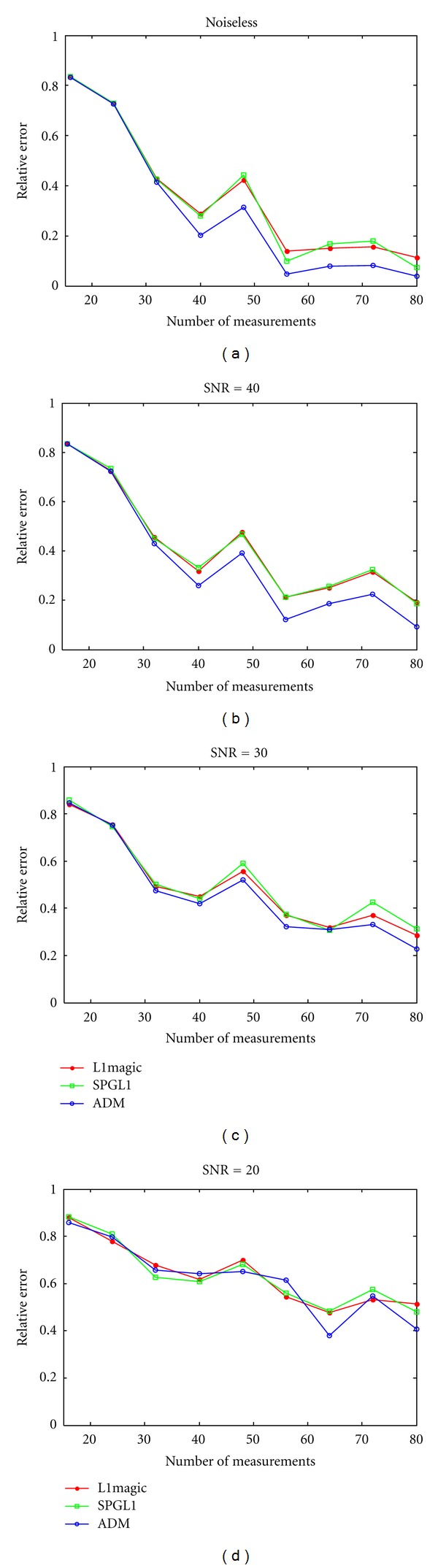
Relative errors for different CS reconstruction algorithms at different positions for a uniform distribution at a 90-degree curve. (a) Noiseless observation; noisy observation with (b) SNR = 40 dB; (c) SNR = 30 dB; (d) SNR = 20 dB.

Figure 4 shows the tendency chart of relative errors in reconstructed photoacoustic images obtained with these three CS algorithms. It is clear that the ADM algorithm is efficient and robust. In particular, the proposed algorithm cannot only use fewer measurements to obtain better performance, but also reduce relative errors faster than other tested algorithms. However, while keeping the noise level at SNR = 20 dB, the ADM algorithm sometimes reduced relative errors slower than the other two algorithms.

## 4. Conclusion

We presented a novel fast algorithm for PAI using a small number of angles. The proposed algorithm is based on the CS theory. Numerical simulations showed that our algorithm produced better images than FBP and other state-of-the-art CS algorithms. Moreover, the proposed algorithm has been shown to be robust to noise in limited-view imaging. Ongoing work includes a more thorough experimental evaluation of ADM.

## Figures and Tables

**Table 1:** Numerical results for L1magic, SPGL1, and ADM methods on PAI images with different sampling angles and 64 Fourier samples per angle uniformly covering the 90-degree view.

Positions	Experimental								
	Iterations			CPU time (seconds)			SNR (dB)		
	Magic	SPG	ADM	Magic	SPG	ADM	Magic	SPG	ADM
16	67	340	88	471.2	16.3	5.5	−3.8	−3.6	−3.5
24	75	422	73	692.1	28.7	3.4	0.7	0.9	3.9
32	77	345	68	831.5	29.5	7.9	3.3	3.4	3.6
40	78	350	58	877.5	37.5	6.6	11.1	12.1	13.4
48	80	337	53	1280.7	43.2	9.3	11.1	11.7	13.8
56	79	317	46	1294.2	45.3	4.1	20.3	24.6	28.6
64	79	429	43	1179.6	64.5	5.0	20.4	22.1	25.9
72	81	367	46	1711.9	66.4	6.0	21.2	22.9	28.7
80	84	366	42	1580.9	68.7	11.4	22.7	29.1	29.7

Average				1102.2	44.5	6.6			
